# In Vitro Oxidative Crosslinking of Recombinant Barnacle Cyprid Cement Gland Proteins

**DOI:** 10.1007/s10126-021-10076-x

**Published:** 2021-10-29

**Authors:** Robert Cleverley, David Webb, Stuart Middlemiss, Phillip Duke, Anthony Clare, Keiju Okano, Colin Harwood, Nick Aldred

**Affiliations:** 1 School of Natural and Environmental Sciences, Newcastle University, Newcastle upon Tyne, NE1 7RU UK; 2 Biosciences Institute, Newcastle University, Newcastle upon Tyne, NE2 4AX UK; 3 Defence Science and Technology Laboratory, Dstl Porton Down, Salisbury, SP4 0JQ UK; 4 Department of Biotechnology, Akita Prefectural University, Akita, Japan; 5 School of Life Sciences, University of Essex, Wivenhoe Park, Colchester, CO4 3SQ UK

**Keywords:** Barnacle, Cyprid, Adhesion, Cement, Recombinant, Lysyl oxidase

## Abstract

Barnacle adhesion is a focus for fouling-control technologies as well as the development of bioinspired adhesives, although the mechanisms remain very poorly understood. The barnacle cypris larva is responsible for surface colonisation. Cyprids release cement from paired glands that contain proteins, carbohydrates and lipids, although further compositional details are scant. Several genes coding for cement gland-specific proteins were identified, but only one of these showed database homology. This was a lysyl oxidase-like protein (lcp_LOX). LOX-like enzymes have been previously identified in the proteome of adult barnacle cement secretory tissue. We attempted to produce recombinant LOX in *E. coli*, in order to identify its role in cyprid cement polymerisation. We also produced two other cement gland proteins (lcp3_36k_3B8 and lcp2_57k_2F5). lcp2_57k_2F5 contained 56 lysine residues and constituted a plausible substrate for LOX. While significant quantities of soluble lcp3_36k_3B8 and lcp2_57k_2F5 were produced in *E. coli*, production of stably soluble lcp_LOX failed. A commercially sourced human LOX catalysed the crosslinking of lcp2_57k_2F5 into putative dimers and trimers, and this reaction was inhibited by lcp3_36k_3B8. Inhibition of the lcp_LOX:lcp2_57k_2F5 reaction by lcp3_36k_3B8 appeared to be substrate specific, with no inhibitory effect on the oxidation of cadaverine by LOX. The results demonstrate a possible curing mechanism for barnacle cyprid cement and, thus, provide a basis for a more complete understanding of larval adhesion for targeted control of marine biofouling and adhesives for niche applications.

**Supplementary Information:**

The online version contains supplementary material available at 10.1007/s10126-021-10076-x.

## Introduction

Barnacles are well-known for their ability to remain attached throughout their adult life. Hydration and contamination of the adhesive interface, and the high ionic strength of seawater, present significant challenges for synthetic adhesives in similar settings. Barnacles, however, can live for many years and throughout this period their adhesive bond survives biodegradation and exposure-immersion cycles, and accommodates growth of the animal, adapting to the morphology of the substratum. The adhesive bond must be sufficiently strong to resist removal by predators and the local hydrodynamics, and also compatible with a diverse range of natural surfaces that may be selected by the settling larva, the cyprid (Aldred and Clare 2008). Several decades of research into the adhesion of adult barnacles have improved understanding of their dynamic adhesion process with a view to preventing barnacle adhesion on artificial structures at sea (antifouling) and/or developing synthetic adhesives with niche capabilities (bioadhesion). However, the fundamentals of barnacle adhesion remain poorly understood (Liang et al. 2019; Davey et al. 2021).

Adult barnacle adhesion is complex in terms of its composition, the morphology of the adhesive interface and the sequential process by which the interface is formed (Schultzhaus et al. 2020). Despite substantial progress, it is clear that significant research will be required before a working understanding of adult barnacle adhesion can be claimed. Interestingly, the cyprid permanent adhesive system has received relatively little attention (Walker 1971; Okano et al. 1996, 1998; Ödling et al. 2006; Aldred et al. 2013, 2020; Gohad et al. 2014; Yan et al. 2020). Although cyprids are small (typically 0.5–1 mm in length depending on the species), there are compelling reasons to focus attention on them. They are arguably the most relevant life stage of barnacles in the context of biofouling, being responsible for initial surface selection and adhesion. Improved understanding of how they choose a substrate and stick permanently to it could significantly benefit the development of fouling-control technologies. The cyprid adhesion system is also, ostensibly, more tractable for study than that of the adult barnacle. Adhesion of adult barnacles is a dynamic process that involves perforation and formation of new cuticle, the release of various fluids of unknown origin and specific composition, and specialised secretory cells with associated duct networks (Fears et al. 2018). The adhesive interface is located beneath the barnacle (Essock-Burns et al. 2017), making collection of adhesive material problematic (Dickinson et al. 2009; Kamino 2010; Schultzhaus et al. 2019). Anatomically, the origins of the adhesive precursors inside the barnacle are not well understood (Schultzhaus et al. 2020), so RNA-Seq and/or proteomics-based analyses of the production apparatus are difficult to target for acorn barnacles (Alm Rosenblad et al. 2021; Davey et al. 2021; Dominguez-Pérez et al. 2021).

Although much smaller, the larval adhesive system presents fewer challenges. The location of adhesive production is clear, occurring within a pair of large cement glands inside the body of the cyprid. The glands contain two cell types (the α and β cells) that are well-defined, easily identifiable (Walker 1971) and contain different cement precursors including proteins, carbohydrates and lipids (Aldred et al. 2020). While proteins are present in both cell types, lipids and chitin are restricted to the β cells. The contents of these glands are released once a suitable surface for attachment has been selected, embedding the terminal antennular segments in a relatively large adhesive plaque (Walker 1971). The cyprid permanent adhesive, or cement, is therefore amenable to investigation providing that techniques can utilise the tiny quantities of sample available per animal. Although this may be true for some imaging and spectroscopic methods, it is rarely the case for chromatographic or other bioanalytical techniques. Experiments designed to understand the roles and interactions of specific cement components will always, therefore, require more material than can practically be collected from larvae.

An alternative approach is to identify proteins of interest from within the cement glands, using techniques such as RNA-Seq and high-resolution proteomics that can tolerate small quantities of sample, followed by recombinant production of those proteins in heterologous expression systems. In principle, larger quantities of recombinant proteins would then be available for investigation. This approach assumes that the proteins are produced in an authentic state by the heterologous system, retaining key functionality. If the native protein is heavily disulfide bonded, glycosylated or otherwise post-translationally modified, this may not be the case.

The present study identified a range of uncharacterised proteins within the cyprid cement gland and attempted their recombinant production in *Escherichia coli*. The central aim was to produce sufficient quantities of proteins to supply hypothesis-driven experiments that may reveal the possible roles of those proteins in vivo. Of the proteins identified as being enriched in the cement gland, three were selected for recombinant production based upon their abundance, storage location and biochemical nature. The first, a 57 kDa protein with 56 lysine residues and no homologues in public databases, was a potential substrate for a second protein, a putative lysyl oxidase (LOX). A third protein of 36 kDa was included on the basis of its abundance and storage location.

Lysyl oxidases are enzymes of particular interest in barnacle adhesion research, as they have been discovered at the adult barnacle adhesive interface (So et al. 2017) and in a cyprid transcriptome (Yan et al. 2020). A role for LOX in cyprid adhesion could not only provide insight into the curing mechanism of cyprid cement, but also provide a direct link between the adult and cyprid adhesion systems. Lysyl oxidases are instrumental in the formation and maintenance of eukaryotic extracellular matrices (ECM). They achieve this by covalently crosslinking the lysine residues present in collagen and elastin, the primary structural proteins of the ECM. LOX catalyses the oxidative deamination of the ε-amino group of lysine residues, producing reactive aldehydes that condense with neighbouring aldehydes or ε-amines to form strong intermolecular crosslinks (Zhang et al. 2018). Although a role for LOX (So et al. 2017) and oxidases in general (Dickinson et al. 2009) has been mooted for adult barnacle adhesion previously, a functional role in adhesion has not been demonstrated and the process of cuticle formation, that occurs concurrently with adult barnacle adhesion, complicates interpretation of those observations. The cyprid cement gland and the secreted adhesive material are distinct from the locations of cuticle formation in cyprids, and the cyprid adhesion system may therefore provide a convincing platform from which to demonstrate a possible role for LOX in barnacle adhesion. If LOX is involved in cyprid adhesion, this could imply that cyprid adhesion evolved from a process related to ECM formation.

## Methods

### Identification of Cement Gland-Specific Proteins

Cyprids of *Megabalanus rosa* were provided by Dr. Yasuyuki Nogata (Environmental Research Laboratory of the Central Research Institute of Electric Power Industry, Japan). A variety of approaches were used to determine the amino acid (AA) sequences of the three cement gland-specific proteins that are the focus of this paper (K. Okano et al. in preparation). Briefly, cement glands were dissected from *M. rosa* cyprids using electrochemically etched tungsten needles (Okano et al. 1996) and used for protein and cDNA analysis. Cement gland-specific bands of 36 kDa and 57 kDa, separated by SDS-PAGE, were digested and partially sequenced using Edman degradation (Aldred et al. 2020; Okano et al. in preparation). Using these partial amino acid sequences as probes, full-length cDNA clones were selected from a *M. rosa* cement-gland full-length cDNA library (K. Okano et al. in preparation). Two partial sequences for a lysyl oxidase-like protein were discovered in the cement gland-specific expressed sequence tags, produced by suppression subtractive hybridisation (K. Okano et al. in preparation). The full-length cDNA sequences of the LOX-like protein were obtained using 5′ and 3′ RACE (lcp_LOX: DDBJ accession number, LC596875). Similarly, the cDNA sequences of the 57 kDa (lcp2_57k_2F5: DDBJ accession number, LC596868) and 36 kDa (lcp3_36k_3B8: DDBJ accession number, LC596871) proteins were obtained. The resulting protein sequences were used in this study for the in silico design of *E. coli* codon-optimised versions of lcp3_36k_3B8, lcp2_57k_2F5 and Lcp_LOX, using the JCAT online codon optimisation tool (Grote et al. 2005). The codon-optimised sequences are located in Supplemental 1.

### Cloning, Expression and Purification of Cement Gland Proteins

#### Expression Plasmid Construction

Initial codon-optimised constructs for bacterial expression of lcp2_57k_2F5, lcp3_36k_3B8 and lcp_LOX with N-terminal His_6_ tags pET28a57k, pET28a36K and pET28aLOX were obtained from DNA 2.0 (now ATUM, Newark, California). The pET28aLOX construct encodes amino acids 19–515 of the lcp_LOX preprotein in frame with an N-terminal His_6_tag and thrombin cleavage site; amino acids 1–18 of lcp_LOX, which were omitted, correspond to the signal peptide. The final construct used for 36 K expression, pSF014-SUMO-36 K, was derived from a *Nco*I-*Xho*I fragment of plasmid pSF014-SUMO-DnaA (provided by Prof. Heath Murray, Biosciences Institute, Newcastle University) generated by PCR amplification with primers SF014DnaA5 and SF014DnaA3 (Table 1). This fragment was ligated with a *Nco*I-*Xho*I fragment incorporating the 36 K gene, generated by PCR using the template pET28a36K and primers 36K_5 and 36K_3 (Table 1).

**Table 1:** Primers used in the cloning of cement gland protein genes

Name	Sequence
SF014DnaA5	GGACTACCATGGCGCCACCAGTCTGGTGCAGCATTGC
SF014DnaA3	CGACAGCAATATTCGGGAACTCG
36K_5	CGTGTACCATGGGCACATACTCTCGTGTTTCTCC
36K_3	CGTCTACTCGAGCTTTGTTAGCAGCCGGATCTC
SH91_5	GAGATATACATATGCCATTAACGCCAAATGATATTCACAACAAGACG
SH91_3	GCATCACTCGAGTGGAGCCACCCGCAGTTCGAAAAATAAGG
57K_5	CGATAGCATATGGGCAGCAGCCATCATCATC
57K_3	GCTATCCTCGAGTCATTAGCCTTTTGTTGTTGTAGGGAAGCC
57KmutSTOP5	CCTACAACAACAAAAGGCTCAGGACTCGAGTGGAGC CACC
57KmutSTOP3	GGTGGCTCCACTCGAGTCCTGAGCCTTTTGTTGTTG TAGG
5NcoILox	CGAGTACCATGGGCCAAAACCGTGATTCTTTCGATTTC
3XhoILox	GCAGTACTCGAGGATCATTACAGCGGAGCGTTACG

The final construct used for 57 K expression, pNHis57KStrepII, was derived from a *Nde*I-*Xho*I fragment generated by PCR with primers SH91_5 and SH91_3 from template plasmid pSH91 (Oliva et al. 2010; provided by Dr Sven Halbedel, Robert Kock Institute). This was ligated with a *Nde*I-*Xho*I fragment produced by PCR from template pET28a57K and primers 57K_5 and 57K_3. Two STOP codons between the 57 K and StrepII tag coding sequences were subsequently deleted with codons encoding a SG linker sequence using a QuickChange mutagenesis with primers 57KmutSTOP5 and 57KmutSTOP3 (Table 1).

The construct for expressing LOX as an MBP fusion protein was generated by subcloning into the NcoI and XhoI restriction sites of the pMAT11 plasmid (Peränen et al. 1996), a gift from Marko Hyvönen and Johan Peränen (Addgene plasmid # 112,592; http://n2t.net/addgene:112592; RRID:Addgene_112592). The appropriate fragment, encoding barnacle LOX residues 19–515 with flanking NcoI and XhoI restriction sites, was generated by PCR from the pET28aLOX plasmid using primers 5NcoILOX and 3XhoILOX (Table 1).

#### lcp3_36k_3B8 Expression and Purification

All cultures were grown in Luria broth containing 100 µg/mL carbenicillin. BL21(DE3) was transformed with plasmid pSF014-SUMO-36 K and a 100-mL culture was grown to an OD_600_ of c. 0.8 at 37 °C. Twenty millilitres of this culture was then centrifuged for 5 min at 4,000* g* and the pellet was transferred to 1L of fresh media. Upon reaching an OD_600_ of 0.8, the 1-L culture was induced overnight at 20 °C with 0.4 mM IPTG.

The pellet from 2 L of culture was resuspended to a final volume of 60 mL in buffer A (50 mM Tris pH 8 300 mM NaCl 20 mM Imidazole) containing a Roche COMPLETE EDTA-free protease inhibitor cocktail tablet, 400 units of DNAase and 30 mg of lysozyme. After sonication, the resulting lysate was centrifuged for 20 min at 19,000* g* and the supernatant was loaded onto a 5-mL HisTrap IMAC cartridge (GE Healthcare) at 2 mL/min. After extensive washing with buffer A, the protein was eluted with a 30-mL gradient increasing to a final imidazole concentration of 500 mM and a final glycerol concentration of 10%. Fractions containing the His_14_-SUMO-36 K fusion protein were pooled, diluted four-fold with 50 mM Tris pH 8 300 mM NaCl and 10% glycerol then incubated overnight at 4 °C with 0.3 mg of SUMO protease. LDAO (N-dodecyl-N,N-dimethylamine N-oxide, Sigma) detergent was then added to a final volume of 0.1% w/v and the solution passed through a 0.2-micron syringe filter. The NaCl concentration was then reduced to < 10 mM by several cycles of dilution/reconcentration using 50 mM Tris (pH 8), 10% glycerol, 0.1% LDAO (36K_IEX_Buffer_A) for dilution and a 15-mL centrifugal concentrator with a 30 kDa molecular weight cut-off filter. The buffer-exchanged 36 kDa protein was then passed at a flow rate of 0.5 mL/min over a 1-mL HiTrap QFF (GE Healthcare) cartridge equilibrated in 36K_IEX_Buffer_A. Flow-through fractions containing the 36 K protein were pooled and concentrated in a 15-mL 30 kDa cut-off concentrator to a concentration of c. 2 mg/mL, and then small aliquots were frozen in liquid nitrogen and stored at −80 °C.

#### lcp2_57k_2F5 Expression and Purification

Strain SHuffle® T7 Express, transformed with plasmid pNHis57KStrepII, was used to inoculate 100-mL Luria broth containing 100 µg/mL carbenicillin at 37 °C. Upon reaching an OD_600_ of approximately 0.8, 35 mL of this culture was centrifuged for 5 min at 4000* g* and then the pellet was transferred to 1 L of Terrific broth media containing 100 µg/mL carbenicillin. Upon reaching an OD_600_ of 0.6–0.8, the culture was cooled and then induced overnight at 20 °C with 0.4 mM IPTG.

The pellet from the 2 L culture was resuspended in buffer A, containing a Roche COMPLETE protease inhibitor cocktail tablet and 30 mg lysozyme, to a final volume of approximately 40 mL. The cell suspension was subjected to 4 cycles of freeze–thaw lysis, using liquid nitrogen for freezing and a 37 °C water bath for thawing. Four hundred units of DNAase were then added and the lysate was centrifuged at 23000* g* for 20 min. The supernatant was then supplemented with MgCl_2_ to a concentration of 4 mM, filtered through a 0.4-micron syringe filter and loaded onto a 5-mL HisTrap IMAC cartridge (GE Healthcare) at 1 mL/min. After extensive washing with buffer A, the 57 K protein was eluted over a 25 mL gradient to a final imidazole concentration of 500 mM.

Pooled fractions were concentrated to 10 mL and combined with a 1 mL bed volume of Streptactin-XT® (IBA) Superflow® High Capacity beads in a 15-mL-centrifuge tube. After 2 h of constant inversion-mixing at 4 °C, the mixture was filtered through a 20-mL plastic column and the resin bed then washed with 5 applications of 1 mL of buffer A containing 500 mM imidazole. The 57 K protein was eluted with 100 mM Tris (pH 8), 300 mM NaCl, 50 mM biotin and 1 mM EDTA. Fractions were pooled, diluted tenfold with LOX_assay_buffer (50 mM sodium borate pH 8,10 mM CaCl_2_) and then dialysed twice against 1 L LOX_assay_buffer. Finally, the protein was concentrated to 4 mg/mL, frozen in small aliquots in liquid nitrogen and stored at −80 °C.

#### lcp_LOX Expression and Purification

The pET28aLOX construct was co-transformed with plasmid pMJS205 (Matos et al. 2014; provided by Lloyd Ruddock) into strain SHuffle^®^ T7 Express and transferred to LB media containing 50 μg/mL kanamycin and 34 μg/mL chloramphenicol. Upon reaching an OD_600_ of 1, the culture was induced with 1 mM IPTG and incubated for a further 3 h at 37 °C before harvesting by centrifugation and storing the cell pellet at −80 °C. The plasmid pMJS205, which expresses yeast disulphide bond isomerase and sulphydryl oxidase proteins, has been shown to increase the solubility of disulphide-bonded proteins when expressed in *E. coli* (Gaciarz et al. 2017), but the use of pMJS205 in combination with SHuffle^®^ T7 Express did not enable successful downstream purification of lcp_LOX from the soluble fraction of *E. coli* lysates.

Lcp_LOX was therefore purified from urea-resolubilised inclusion bodies. A total of 2.6 g of frozen cell pellet was resuspended to 30 mL final volume in buffer A containing 0.5 mL of SIGMA protease inhibitor cocktail (catalogue number P8849) and 0.1 mg DNAaseI. After gentle lysis with a sonicator probe, the cell suspension was centrifuged at 23,000 *g* for 20 min at 4 °C. The pellet was then resuspended to 35 mL final volume in 20 mL buffer A supplemented with 2 M urea and 0.25 mL SIGMA protease inhibitor cocktail (catalogue number P8849) and the mixture then centrifuged again at 23,000 *g* for 20 min at 4 °C. The pellet was then resuspended in 30 mL of 50 mM Tris pH 8 300 mM NaCl 10 mM imidazole 8 M urea (Urea_buffer) containing 1 mM DTT and stirred for 1 h. The mixture was then filtered through a 0.45-micron pore size syringe filter and loaded onto a 5-mL HisTrap IMAC cartridge (GE Healthcare) at 1.5 mL/min. After washing the column with > 100 mL of Urea_buffer, the lcp_LOX was eluted from the column with Urea_buffer containing 500 mM imidazole. The eluted protein, at 6 mg/mL concentration by A_280_, was then incubated at 30 °C for 1 h with 2 mM TCEP and 5 mM EDTA and then diluted ten-fold into 20 mM NaHEPES pH 7 2% sarkosyl and dialysed versus 20 mM NaHEPES pH 7 2% sarkosyl 0.2 mM CuCl_2_ 0.1 mM CaCl_2_ 0.5 mM PMSF for 4 h and then dialysed overnight versus 20 mM NaHEPES pH 7 0.1 mM CaCl_2_ 0.2 mM CuCl_2_ 0.5 mM PMSF. The refolded protein was finally dialysed twice versus 20 mM potassium phosphate pH 7.3 5 mM NaCl 0.002% sarkosyl 0.25 mM PMSF. The refolded protein was then concentrated in a 30 kDa molecular weight cut-off centrifugal concentrator to 0.8 mg/mL, flash frozen in liquid nitrogen and stored at −80 °C.

#### MBP-lcp_LOX Expression and Purification

The MBP-lcp_LOX construct was expressed in strain SHuffle^®^ T7 Express in Terrific Broth as described in the *lcp2_57k_2F5 expression and purification* section. The frozen cell pellet was thawed and resuspended to 40–45 mL final volume in buffer A supplemented with 1.4 mL Roche EDTA-free COMPLETE protease inhibitor cocktail (from a stock at 25 × manufacturer’s working concentration) and 600 units DNAase. After lysis by gentle sonication, the lysate was centrifuged at 23,000* g* for 20 min at 4 °C and the supernatant was then filtered through a 0.45-micron pore size syringe filter before loading onto a 5-mL HisTrap IMAC cartridge (GE Healthcare) at 1.2 mL/min. After extensive washing of the column with buffer A, the MBP-lcp_LOX protein was eluted from the column with a 30 mL gradient of 40–500 mM imidazole. Fractions containing the MBP-lcp_LOX protein were pooled and prepared for activity assays using a dialysis protocol similar to that used by Smith et al. (2016) for solubility-tagged human LOX constructs. Pooled fractions of MBP-lcp_LOX were dialysed overnight against 20 mM Tris pH 8 300 mM NaCl 1 mM EDTA, then for 5 h against 20 mM Tris pH 8 300 mM NaCl (buffer C), then overnight against buffer C supplemented with 0.5 mM CuSO_4_ and then finally for 4 h again versus buffer C.

### Characterisation Of Recombinant Cement Gland Proteins

#### Surface Plasmon Resonance

Nonspecific surface adsorption of lcp3_36k_3B8 and lcp2_57k_2F5 was investigated using surface plasmon resonance. Binding experiments used a Biacore S200 instrument and a buffer of 20 mM Tris pH 8, 100 mM NaCl and 3 mM CaCl_2_ at a flow rate of 10 µL min^−1^. Binding was quantified by the increase in response units on the surface after injecting proteins over the SPR chips at a concentration of 0.2 mg/mL for 3 min and washing the surface with buffer for 5 min. A single chip was used for each protein injection; for each surface, each protein was injected over three independently prepared chips. Gold-coated SPR chips (from SIA kit Au, GE Healthcare) were functionalised using the thiols undecyl thiol, (11-mercaptoundecyl)-N,N,N-trimethylammonium bromide and 11-mercaptoundecanoic acid as described previously (Petrone et al. 2015) to form self-assembled monolayers (SAMs) with CH_3_, NMe_3_^+^ and COO^−^ terminations. These were stored at room temperature under nitrogen until required. Contact angles were measured as the average of three static water contact angles from three 20 µL drops of MilliQ water using a Ramé-Hart contact angle goniometer. The average contact angles for the three SAMs were CH_3_ = 95° ± 2 (SD), NMe_3_^+^  = 48° ± 1 (SD) and COO^−^  = 52° ± 1 (SD).

#### Circular Dichroism

Protein secondary structure was interpreted from CD spectra, recorded on a JASCO J-810 spectropolarimeter with a PTC-4235 Peltier 1019 temperature controller using 1 mm path length quartz cuvettes, at 0.1 mg/mL protein concentration in 10 mM potassium phosphate buffer pH 8. Spectra were recorded at a scan speed of 10 nm^−1^ min^−1^ with a response time of 4 s. The dodecyldimethylaminoxide (LDAO) detergent in the 36 K protein was removed prior to measurements by exchanging the sample into 10 mM potassium phosphate buffer (pH 8) using a Centripure Z-50 desalting column (Generon) with a molecular weight cut off of 25 kDa.

#### Activity of Recombinant lcp_LOX

The activity of refolded lcp_LOX was measured in a 60 μL sample volume in 50 mM sodium borate pH 8 10 mM CaCl_2_ 0.25 M urea (LOX assay buffer 2) containing 8 mM benzylamine with lcp_LOX present at 40 ng/μL final concentration. After incubation for 30 min at 37 °C, the sample was combined with 60 μL of 2U/mL horseradish peroxidase and 20 μM Amplex Red in LOX assay buffer 2 and then 100 μL of the mixture was transferred to a black walled, clear bottomed 96-well microplate and the fluorescence signal measured on a BMG Optima Fluostar plate reader with 544 and 590 nm excitation and emission filters, respectively. The change in fluorescence signal upon incubation with LOX was converted to the amount of pmol hydrogen peroxide generated using a standard curve with standards containing between 100 and 500 pmol hydrogen peroxide. This corresponded to an activity of 0.15 nmol min^−1^ mg^−1^ for the lcp_LOX; by comparison, the activity of human LOXL3, measured in parallel under the same conditions, was 14 nmol min^−1^ mg^−1^. Furthermore, the trace lcp_LOX activity was not inhibited after preincubating lcp_LOX with 5 mM of the known LOX inhibitor (Tang et al. 1983) BAPN before the addition of benzylamine substrate; by contrast, the activity of human LOXL3, measured under the same conditions, was fully inhibited upon preincubation with 1 mM BAPN, and human LOXL3 was therefore used for all experiments reported in the “Results” section. Human LOXL3 was obtained from R&D Systems (Minneapolis, MN). This was supplied by the manufacturer at a protein concentration of 0.3 mg/mL in a buffer of 25 mM MES pH 6, 500 mM NaCl; the same buffer was used to dilute the protein to 0.1 mg/mL, and small aliquots were frozen in liquid nitrogen and stored at −80 °C.

#### Analysis of Substrate Protein Interactions

To measure the turnover of recombinant protein substrates by human LOXL3, samples (6 µL volume) of human LOXL3 in LOX_assay_buffer were incubated in a PCR machine at 37 °C for 30 min in 200-µL PCR tubes with LOXL3 at 20 ng/µL concentration and then combined with 6 µL of 2 × substrate mix (2.5 U/mL horseradish peroxidase, 40 µM Amplex Red in LOX_assay_buffer). Ten µL of this mixture was then transferred to a Corning 4154 384-well fluorescence microplate, and the fluorescence was measured on a BMG Optima Fluostar plate reader with 544 and 590 nm excitation and emission filters, respectively. The amount of hydrogen peroxide generated was calculated from the difference between the end point of fluorescence signals of identical samples incubated in the presence and absence of LOX. This fluorescence signal was converted to the amount of hydrogen peroxide using a simultaneously measured calibration curve from samples containing 10–50 pmol hydrogen peroxide.

Turnover of cadaverine by human LOXL3 was measured using the same protocol, except that the substrate protein in the mixture was replaced with 2 mM cadaverine.

To observe the formation of oligomers, recombinant cement gland protein samples of 10 µL volume in LOX_assay_buffer were incubated at 37 °C in 200-µL PCR tubes with LOXL3 at 20 ng/µL concentration. Prior to these interaction assays, lcp3_36k_3B8 was exchanged into LOX_assay_buffer (50 mM sodium borate pH 8, 10 mM CaCl_2_) using Centripure Z-50 desalting columns (Generon) to remove Tris buffer and LDAO detergent. Tris–Glycine (9%) gels were used for SDS-PAGE and blotted onto nitrocellulose membranes. A StrepII-tagged ladder was used for molecular weight calibration (Fisher catalogue number 11892124) of the bands on the blot. StrepII-tagged proteins on the blot were detected with a Streptactin-HRP antibody (IBA Biosciences).

## Results

### General Properties of lcp3_36k_3B8, lcp2_57k_2F5 & and lcp_LOX

**Fig. 1:**
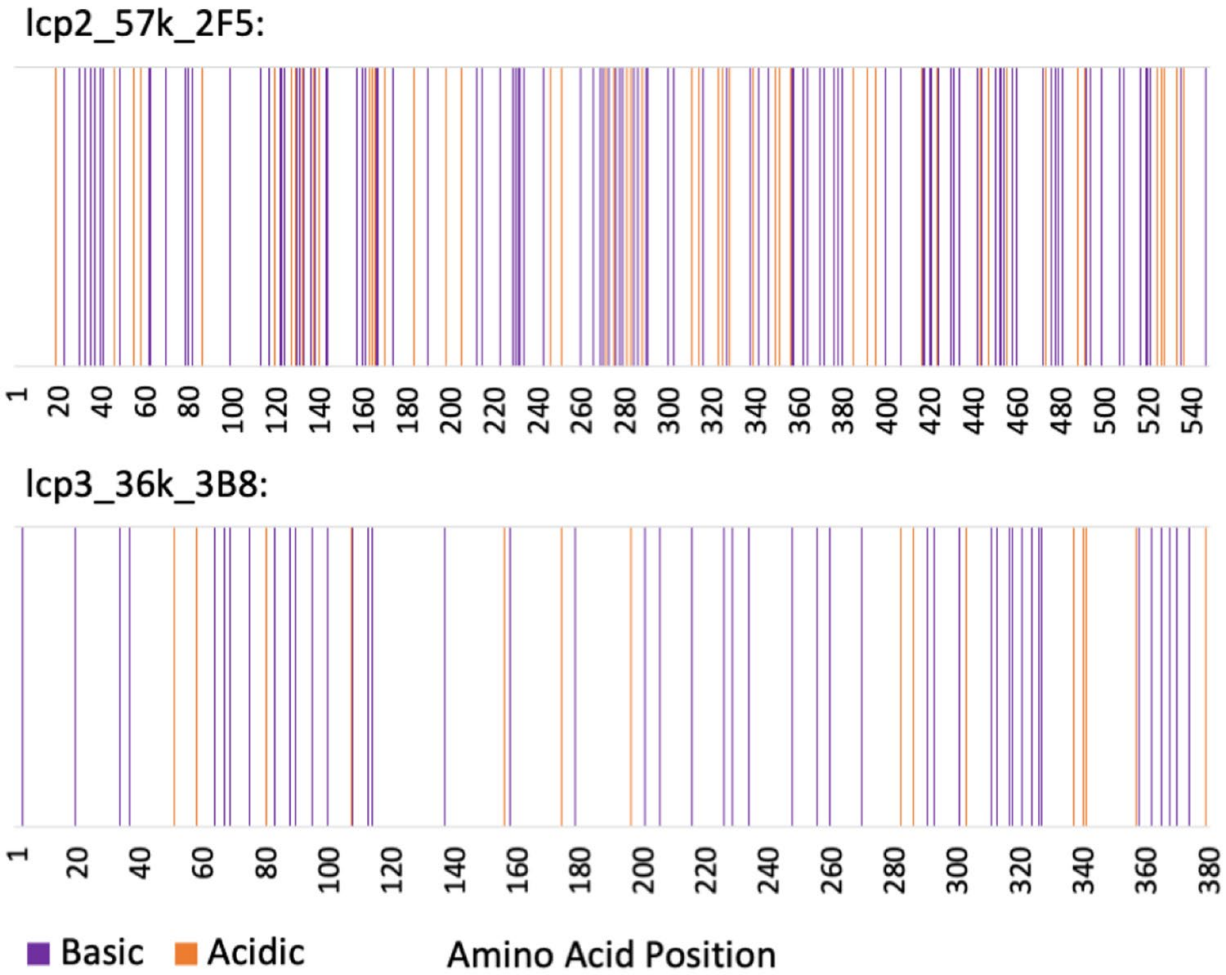
The distribution of charged amino acid residues across the primary structure of lcp2_57k_2F5 & and lcp3_36k_3B8 cement gland-specific proteins

Three proteins of interest were identified for recombinant expression in this study. The first, lcp3_36k_3B8, was 380 amino acid (AA) residues in length and was highly basic with an EMBOSS Pepstats (EMBL-EBI) predicted pI of 11.9. Of the positively charged amino acids, this protein contained only 0.8 mol% lysine and 1.3 mol% histidine, but a remarkable 10.0 mol% arginine (3, 5 and 38 residues respectively) (Table 2). With a positive grand average of hydropathy (GRAVY) value, lcp3_36k_3B8 was predicted to be hydrophobic. In contrast, lcp2_57k_2F5 was predicted to be hydrophilic based on GRAVY (Table 2). It was 548 AA residues in length and also positively charged at neutral pH, with a pI of 10.9. It contained 10.2 mol% lysine, 0.9 mol% histidine and 8.1 mol% arginine (56, 5 and 44 residues, respectively). While both proteins contained small proportions of histidine, lcp3_36k_3B8 was rich in arginine and lcp2_57k_2F5 was rich in both arginine and lysine. lcp2_57k_2F5 contained 12 cysteine residues and, thus, potentially up to six disulfide bonds, whereas lcp3_36k_3B8 contained only three cysteine residues. For both proteins, the acidic and basic residues were distributed relatively uniformly across the primary structure (Fig. 1).

**Table 2:** A summary of relevant sequence information for the 36 kDa and 57 kDa cement gland-specific proteins identified in this study

	**No. Residues**	**pI**	**Aromatic AAs (mol%)**	**Non-polar/polar AAs (mol%)**	**GRAVY**	**Charged (basic/acidic)** **AAs (mol%)**	**Lysine**	**Arginine**	**Cysteine**
36 kDa	380	11.9	10.5	65/35	0.463	16 (12/4)	3	38	3
57 kDa	548	10.9	4.9	45/55	−0.717	28 (19/9)	56	44	12

The lcp_LOX discovered in *M. rosa* (515 AA residues) had 38% sequence identity with human LOX-like 2 (NCBI accession no. 5ZE3_B; Zhang et al. 2018; Supplemental 2) and 67% identity with a LOX-like protein from another barnacle species, *Amphibalanus amphitrite* (NCBI accession no. AQY78507.1; 510 AA residues; So et al. 2017). The *M. rosa* LOX contained an H–H-H putative copper-binding site, as does human LOX. *M. rosa* LOX contained two calcium-binding sites, similar to the human LOX, although in the barnacle enzyme both of these were upstream of the copper-binding site, a feature that, to date, appears to be unique among sequenced LOX-like proteins.

#### Recombinant Production of Cement Gland-Specific Proteins in *E. coli*

Successful approaches were identified for the production of lcp3_36k_3B8 & lcp2_57k_2F5 (see Methods). A significant improvement in yield was achieved for the lcp3_36k_3B8 with the addition of a cleavable N-terminal His_14_-SUMO tag, which dramatically increased the amount of soluble fusion protein. lcp3_36k_3B8 was released from the purified fusion protein by SUMO protease cleavage and could then be separated from the fusion partner in the presence of a mild detergent, LDAO. In the absence of detergent, lcp3_36k_3B8 bound non-specifically to chromatography matrices and other surfaces. The LDAO was removed on a desalting column at the end of the purification without impacting the protein’s solubility.

In the case of the cysteine-rich lcp2_57k_2F5 (Table 1), the level of soluble protein expressed was substantially improved by using the T7 express SHuffle strain, which is amenable to the formation of disulfide bonds in the cytoplasm. Downstream recovery of soluble protein was much more reproducible when using freeze–thaw lysis as opposed to sonication; the particular sensitivity of cysteine-rich proteins to sonication lysis has been previously reported (Stathopulos et al. 2004).

The lcp_LOX was obtained in soluble form by following a previously described refolding protocol (Jung et al. 2003), but was found to be inactive. A fusion protein with an N-terminal MBP (maltose binding protein) tag was produced and partially purified from soluble extracts, without denaturants, but LOX activity was not evident in the purified protein. lcp_LOX was therefore substituted with a commercially available human LOX (LOXL3) in the following experiments. The amino acid sequence of LOXL3 aligns with almost the entire 497 residue sequence of the mature lcp_LOX, with the exception of the first 51 amino acids. The sequence identity between the aligned LOX sequences is 38% and the probability that such an alignment would occur by chance, given by the BLAST expectation value (3 × 10^−80^), is negligible. The human LOXL3 protein was therefore a valid functional surrogate for the barnacle LOX in this proof-of-concept study.

**Fig. 2:**
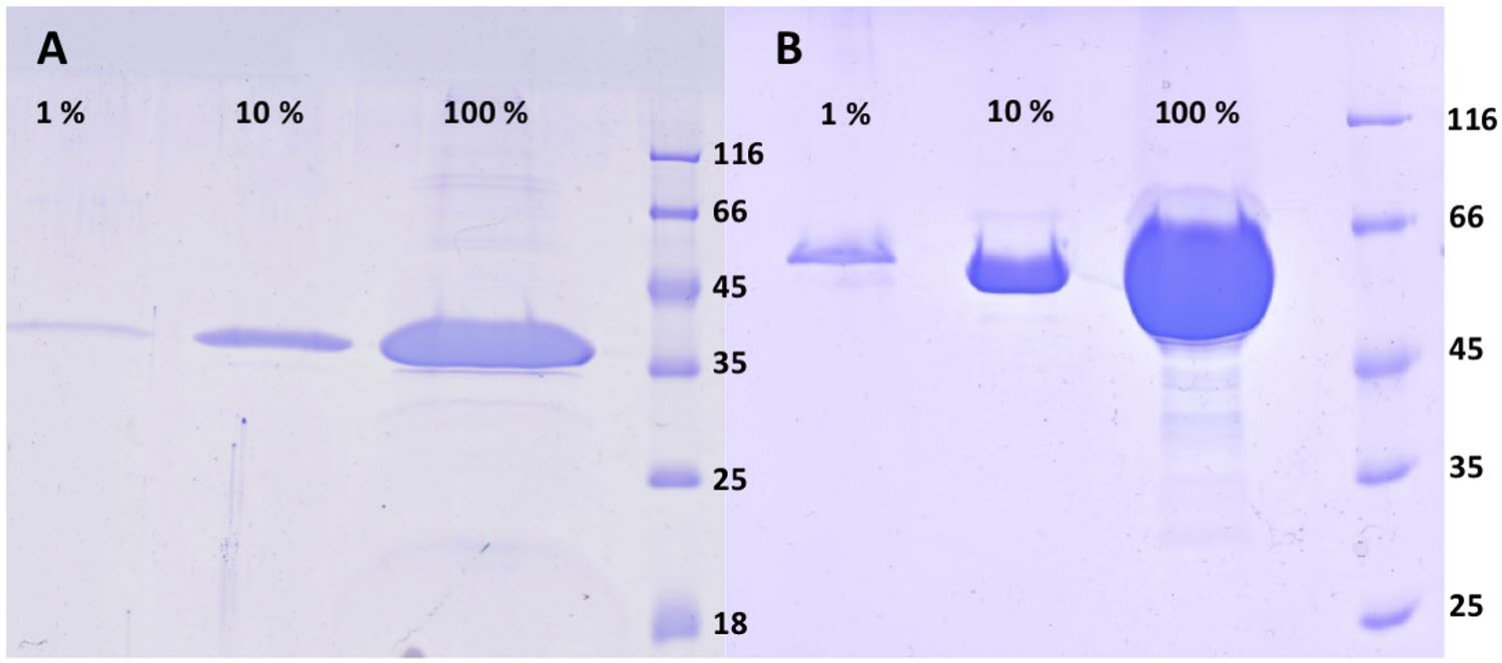
Proteins resulting from recombinant production of **A** lcp3_36k_3B8 and **B** lcp2_57k_2F5 cement gland proteins, separated by SDS-PAGE and stained using Coomassie brilliant blue. Percentage values indicate the quantity of protein loaded relative to the neat sample in the 100% lane. Molecular size markers are measured in kDa

The soluble fractions of lysed cultures were purified and the products visually evaluated by SDS-PAGE (Fig. 2). Although very faint bands of various sizes were visible in the lanes containing the neat samples (100%) of the lcp3_36k_3B8 (Fig. 2A) and lcp2_57k_2F5 (Fig. 2B) cement gland proteins, these were considerably fainter than the target protein band in the 1% lane and, thus, represented less than 1% of the final protein suspension. The soluble protein products were therefore of high purity.

### Nonspecific Adsorption Characteristics of Recombinant lcp3_36k_3B8 and lcp2_57k_2F5 Proteins

Surface plasmon resonance (SPR) was used to investigate the nonspecific adsorption of the two recombinant proteins to three self-assembled monolayers (SAMs). Monolayers with CH_3_, NMe_3_^+^ and COO^−^ terminal groups were produced on gold-coated SPR chips and adsorption experiments were conducted within a Biacore S200 system. Protein adsorbance was compared to fibrinogen and lysozyme as positive and negative standards for nonspecific adsorption, and analysed via 2-way analysis of variance in SPSS v25 (*n* = 3 measurements on all surfaces).

**Fig. 3:**
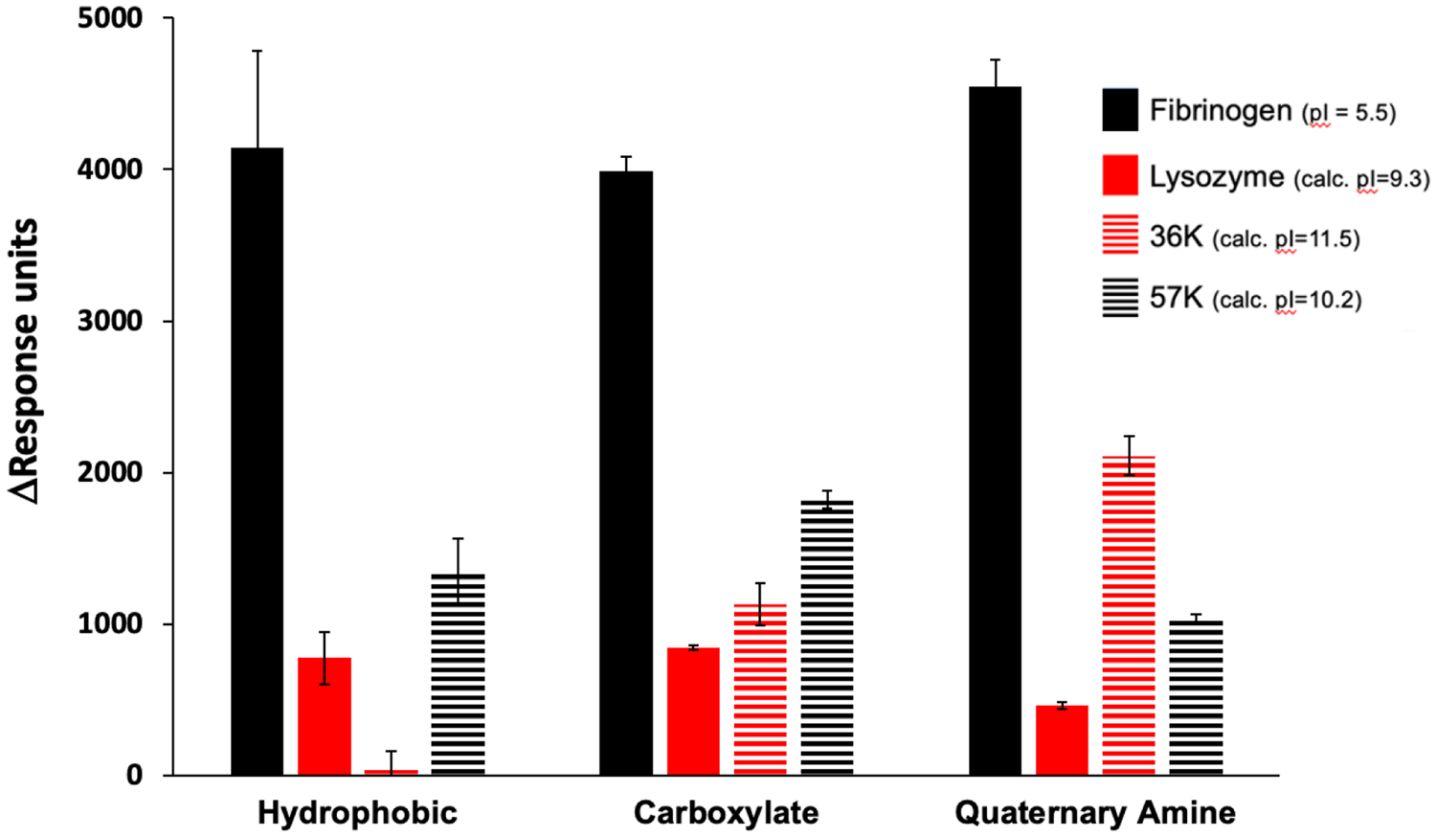
Protein adsorption, reported as mean response units (error bars = one standard error) from SPR experiments (*n* = 3) for the two recombinant cement gland proteins and positive (fibrinogen) and negative (lysozyme) adsorption standard proteins on three self-assembled monolayers with hydrophobic (CH_3_), carboxylate (COO^−^) and quaternary amine (NMe_3_^+^) terminal chemistries

At the physiological salt concentrations and seawater pH used in the experiments, the recombinant proteins were not found to be especially ‘sticky’, relative to fibrinogen (Fig. 3). There were significant overall effects of surface (F_2_ = 4.967, *P* = 0.016) and protein (F_3_ = 160.460, *P* =  < 0.001) in the assay as expected, and a significant interaction between surface and protein (F_6_ = 7.702, *P* =  < 0.001). Thus, the adsorption response varied between proteins and between surfaces, and the response to surface was inconsistent between proteins. A Tukey post hoc test indicated that fibrinogen adsorption was significantly higher than all other proteins (*P* =  < 0.001) and that lysozyme adsorption differed from that of 57 k (*P* = 0.003). No other differences between proteins were significant.

Below its pI of 11.9, lcp3_36k_3B8 should not carry a net negative charge and, therefore, should not show electrostatic affinity to surfaces with net positive charges. However, in this experiment, lcp3_36K_3B8 had elevated adsorption to the quaternary amine SAM, suggesting involvement of factors other than electrostatics in its surface affinity. lcp3_36K_3B8 showed practically no binding to the hydrophobic CH_3_ SAM. The adsorption properties of lcp2_57k_2F5 followed a similar pattern to lysozyme.

### Secondary Structures of the Recombinant lcp3_36k_3B8 & lcp2_57k_2F5

**Fig. 4:**
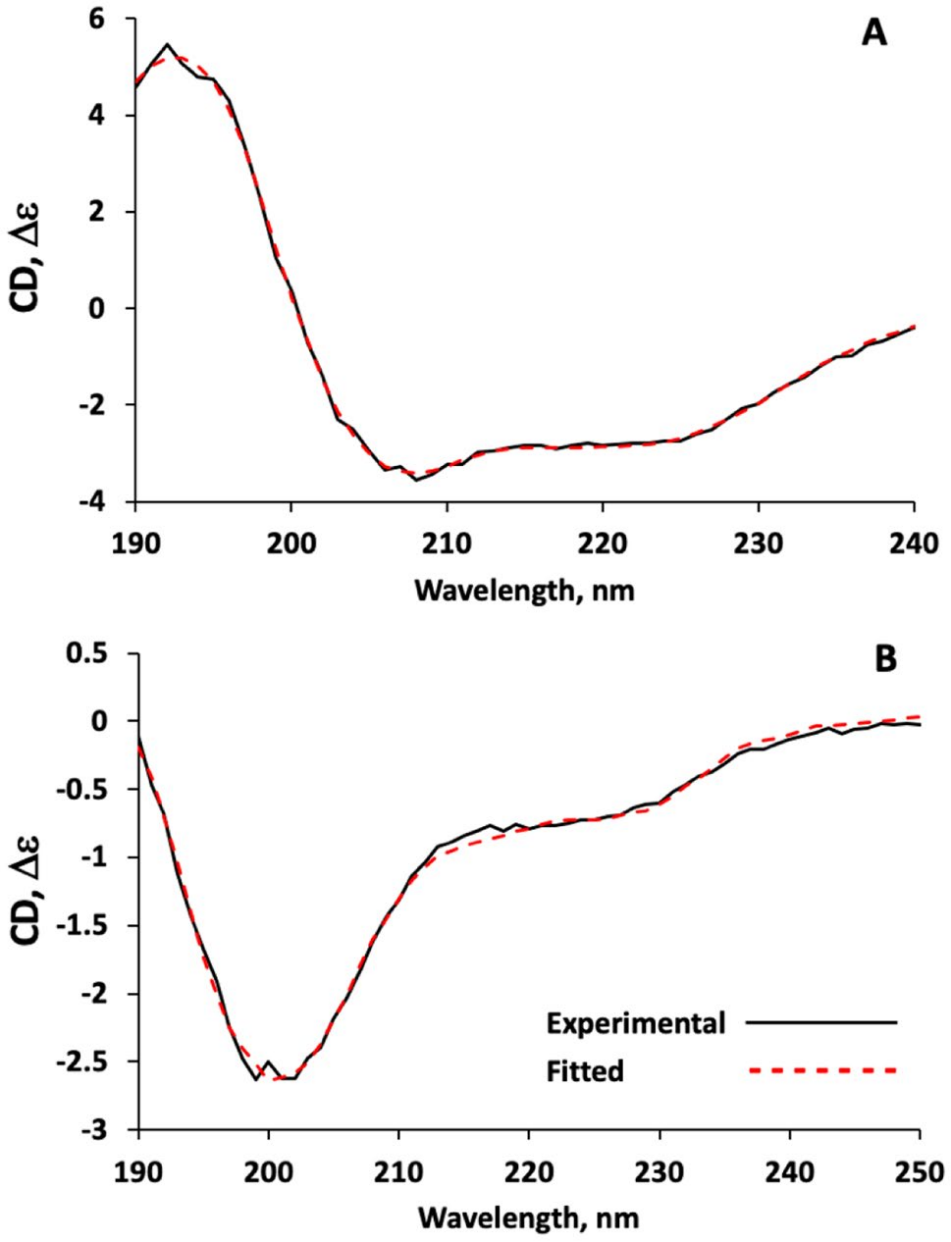
Experimental and fitted circular dichroism spectra for **A** lcp3_36k_3B8 and **B** lcp2_57k_2F5 recombinantly produced cyprid cement gland proteins

The secondary structures of the recombinant cement gland proteins were investigated using circular dichroism (CD). CD spectra showed that there were significant differences between the secondary structures of the recombinant lcp3_36k_3B8 and lcp2_57k_2F5 cement gland proteins. The lcp3_36k_3B8 contained 27% α-helix, 12% β-sheet, 14% β-turn and 47% unclassified regions (Fig. 4A). Analysis of the CD data using the Fold Recognition tool of BeStSel (Micsonai et al. 2018) highlighted several protein families with structural proportions similar to those of lcp3_36k_3B8. Although lcp3_36k_3B8 contained a substantial proportion of α-helix, this content was nevertheless lower than structural predictions had suggested. For example, the Robetta model (https://robetta.bakerlab.org/) predicted > 55% α-helix in lcp3_36k_3B8. It is possible that the 27% α-helix measured by CD could be an underestimate if the 10.5 mol% of aromatic residues (Table 1) of the 36 kDa protein (11 tyrosine residues, 4 tryptophan residues) was located in regions of secondary structure.

The 57 kDa protein, on the other hand, was predominantly composed of β-strands and turns (Fig. 4B) containing only 3% α-helix, 33% β-sheet, 16% β-turn and 47% unclassified regions. It was proposed at the outset that the 57 kDa protein may be a substrate for the LOX, crosslinking itself or lcp3_36k_3B8 into oligomers in a bulk or cohesive role. Thirty three percent of β-sheet and negligible α-helix in the 2° structure of this protein could imply a propensity to form amyloid structures, which would support this function.

### LOX-Induced Crosslinking of lcp2_57k_2F5

**Fig. 5:**
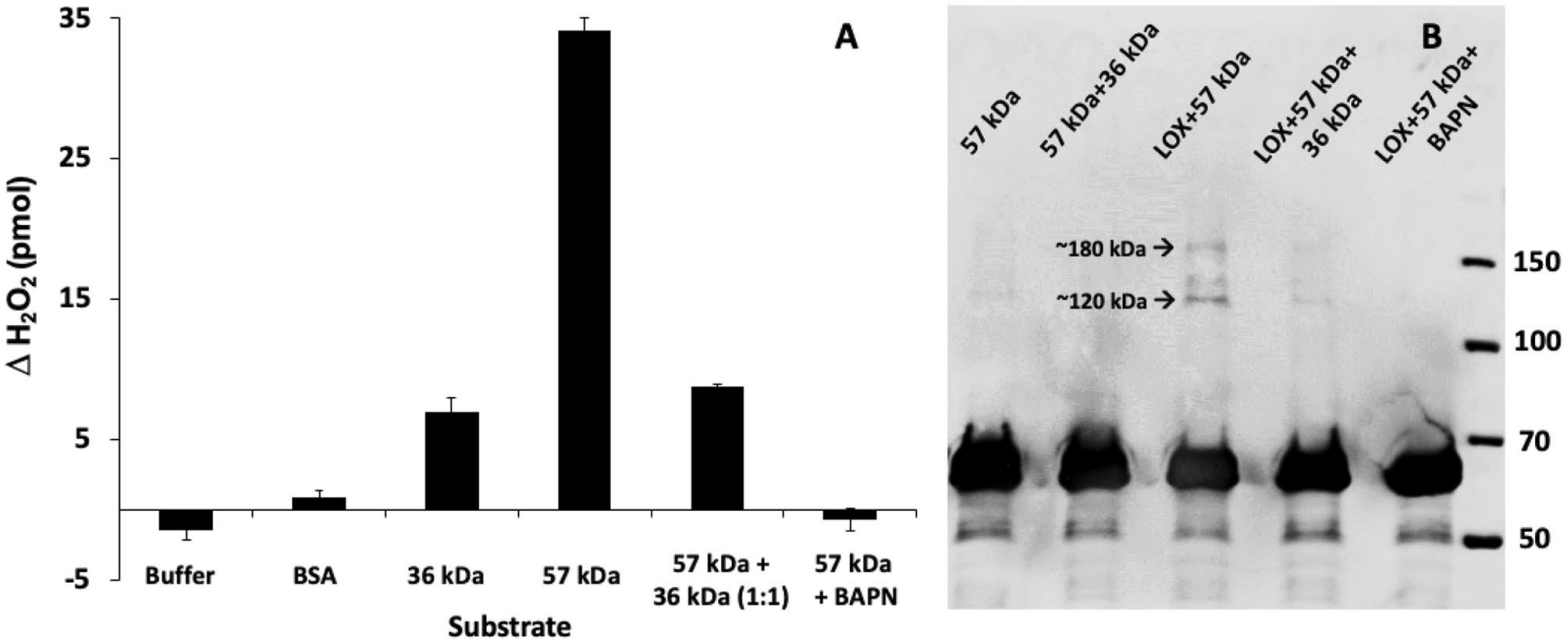
**A** Exposure of the lcp2_57k_2F5 (57 kDa) recombinant cement gland protein to LOXL3 led to oxidation, as indicated by production of hydrogen peroxide in the assay (pmol H_2_O_2_ ± SE). The y-axis shows the amount of H_2_O_2_ released on incubation of 60 pmol of different substrate proteins with LOXL3, calculated from the difference in the H_2_O_2_ levels detected in identical samples after incubation in the presence and absence of 1.6 pmol LOXL3. Bovine serum albumen was not a substrate for LOXL3 and exposure of lcp3_36k_3B8 (36 kDa) to LOXL3 also produced relatively little H_2_O_2_ compared to the lcp2_57k_2F5 substrate. Both lcp3_36k_3B8 and the LOX inhibitor BAPN reduced the oxidation of lcp2_57k_2F5. LOXL3 did not release H_2_O_2_ in the absence of substrate; the very slight reduction in H_2_O_2_ in the ‘Buffer’ sample may be due to background levels of H_2_O_2_ in the assay buffer. Error bars = one standard error. **B** From band migration patterns on a western blot, it was determined that lcp2_57k_2F5 did not spontaneously polymerise with itself (first lane on left), or with lcp3_36k_3B8 (second lane). Exposure to LOXL3 (third lane) produced putative dimers and trimers. Addition of lcp3_36k_3B8 (fourth lane) and BAPN (fifth lane) inhibited this complex formation. The western blot with streptactin-HRP antibody detected the StrepII epitope tag on the C-terminus of the lcp2_57k_2F5 protein. The molecular size markers (far right) are measured in kDa

Having demonstrated that the two recombinant cement gland proteins were not inherently ‘sticky’, in terms of their nonspecific adsorption to CH_3_, NMe_3_^+^ and COO^−^ monolayers, the central hypothesis of the study was addressed; that lcp2_57k_2F5, containing 56 lysine residues, could be a substrate for lysyl oxidase alone or in combination with lcp3_36k_3B8. An in-house assay using production of hydrogen peroxide as a proxy for LOX activity (Fig. 5A) measured only 1 pmol of H_2_O_2_ produced during the in vitro reaction when bovine serum albumen (BSA) was provided as a LOXL3 substrate, despite BSA containing 59 lysine residues per molecule. By comparison, lcp3_36k_3B8, containing only 3 lysine residues per molecule, produced 7 pmol of H_2_O_2_. Clearly distinguished by the assay, however, was the lcp2_57k_2F5 recombinant cement gland protein, which has a similar number of lysines to BSA and yet produced 34 pmol of H_2_O_2_. The activity of LOXL3 on the lcp2_57k_2F5 substrate was abolished completely by the LOX inhibitor β-aminoproprionitrile (BAPN). Surprisingly, addition of lcp3_36k_3B8 to the lcp2_57k_2F5:LOXL3 reaction also provided inhibition, returning the production of H_2_O_2_ close to the level observed for lcp3_36k_3B8 alone, at 9 pmol.

The nature of the lcp2_57k_2F5:lcp_LOX reaction was investigated further by visualising the reaction products using a combination of SDS-PAGE and western blotting (Fig. 5B). The lane containing lcp2_57k_2F5 alone and with lcp3_36k_3B8 showed a single band for lcp2_57k_2F5 of the expected size. However, the lane with reactants lcp2_57k_2F5 and LOXL3 contained additional bands with molecular sizes approximating to dimers (~120 kDa) and trimers (~180 kDa). The inclusion of BAPN in the reaction abolished reactivity entirely while lcp3_36k_3B8 impeded reactivity, reducing the strength of the 120 and 180 kDa bands to barely visible. It was therefore demonstrated that lcp2_57k_2F5 was an authentic substrate for LOX compared with BSA, which, with 59 lysine residues, could plausibly have acted as a substrate. Based on its low but nevertheless significant LOX reactivity (Fig. 5A), lcp3_36k_3B8 could potentially crosslink a LOX-oxidised lcp2_57k_2F5 molecule, but appeared instead to impede its oxidation.

**Fig. 6:**
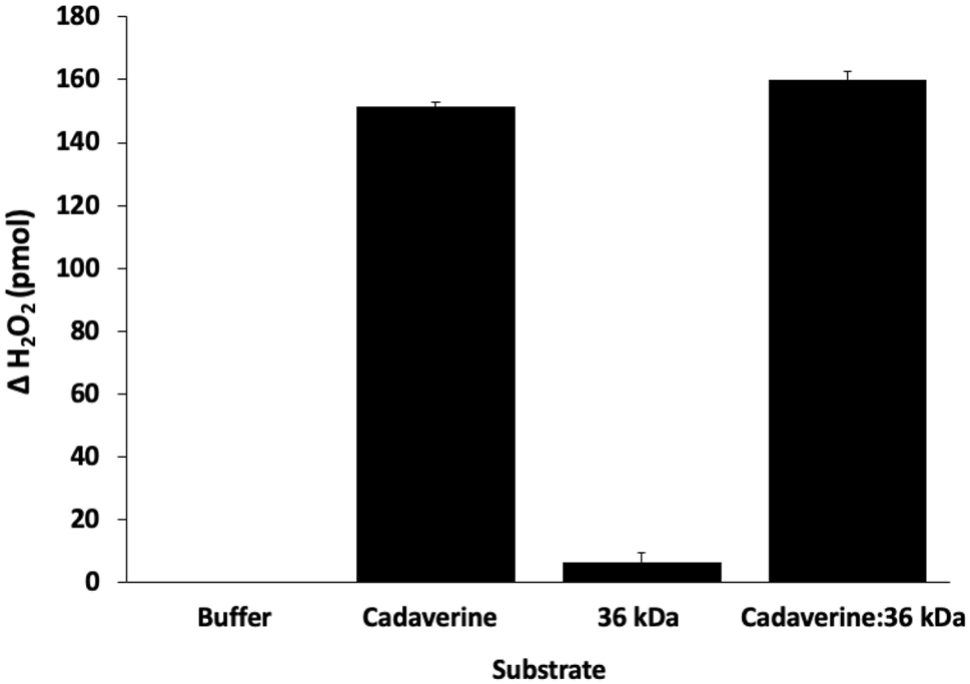
Production of hydrogen peroxide in an assay to monitor the reaction of LOXL3 with a cadaverine substrate, and the lack of inhibitory action by the lcp3_36k_3B8 recombinant cement gland protein. Error bars = one standard error

To examine the specificity of lcp3_36k_3B8 as a LOX inhibitor, the model LOX substrate cadaverine (a decarboxylation product of lysine) was exposed to combinations of LOXL3 and the recombinant lcp3_36k_3B8 cement gland protein (Fig. 6). The well-documented ability of cadaverine to act as a LOX substrate was demonstrated by the production of 151 pmol of H_2_O_2_. As in Fig. 5A, lcp3_36k_3B8 alone produced small, but significant, quantities of H_2_O_2_. Contrary to the results in Fig. 5A, however, addition of lcp3_36k_3B8 to the cadaverine:LOXL3 reaction did not result in inhibition. Rather, the H_2_O_2_ production increased marginally from 151 to 160 pmol.

## Discussion

Interest in biological adhesion has increased over recent years, driven in the case of barnacles by their prominent role in marine biofouling. While knowledge of the broad composition and morphology of barnacle cyprid cement has improved, little progress has been made towards identifying the mechanisms of adhesion and cohesion that could be mimicked in synthetic glues or interfered with by fouling-control technologies. Deep molecular profiling of the cyprid cement glands identified numerous proteins in addition to Mr-lcp1-122 k (Aldred et al. 2020) of which two, 36 kDa and 57 kDa, were abundant in the α and β cells of the cement gland, respectively (K. Okano et al. in preparation). A third protein, a putative lysyl oxidase (lcp_LOX), was also present in the α cells and the abundance of lysine residues in the primary sequence of lcp2_57k_2F5 (Table 1) suggested that it might be a substrate for LOX. Thus modified, lcp2_57k_2F5 could then crosslink with others in a process analogous to the LOX-mediated crosslinking of collagen fibrils in the extracellular matrix. In that process, the ε-amines of lysine residues are converted to Schiff bases by interaction with the lysyl tyrosylquinone (LTQ) cofactor of LOX. Ultimately, α-aminoadipidic-δ-semialdehydes, or allysines, are produced that are highly reactive and form crosslinks with other modified or unmodified lysine residues.

### Production of Recombinant Proteins

In bacterial expression systems, obtaining glycosylation and the correct formation of disulfide bonds are two major issues. This study was ambitious in attempting the production of three moderately large (36 kDa, 57 kDa and 55 kDa [LOX]) proteins of unknown native structure and function. The amino acid sequences of all proteins contained numerous cysteines and, therefore, possible disulfide bonds that would need to be formed correctly for the resulting proteins to retain their intended functionality (3 cysteine residues in lcp3_36k_3B8, 12 in lcp2_57k_2F5 and 22 in lcp_LOX). The protocols described in the methods section produced highly purified, soluble protein from the lcp3_36k_3B8 and lcp2_57k_2F5 sequences; however, lcp_LOX produced by these and other methods was both difficult to solubilise and consistently inactive.

Human LOX contains a copper ion (Cu^2+^) cofactor (Gacheru et al. 1990) and the lysyltyrosine quinone (LTQ) cofactor (Wang et al. 1996) that is formed by a cross-link between lysine 320 and tyrosine 355. The three histidine residues that are known to coordinate with the copper ion (Lopez and Greenaway 2011), and the lysine and tyrosine residues that cross-link to form the LTQ, are all conserved in the cyprid LOX so are presumably required for its catalytic activity. The human pro-LOX is glycosylated at three asparagine residues, two of which are conserved in the N-terminal pro-region of the barnacle LOX. There is also a glycosylation site in the catalytic domain of human LOXL2, which needs to be preserved in order to obtain the soluble LOXL2 from a *Drosophila* expression system (Xu et al. 2013). Such glycosylations would not be produced in bacterial cells. However, in vivo, the pro-region of the LOX protein is cleaved to yield the mature enzyme and our recombinant approach aimed to produce this mature protein without the pro-region, thus circumventing the requirement for glycosylation and cleavage. Although this approach, using *E. coli* expression with subsequent solubilisation and refolding under denaturing conditions, has a basis in the literature (Herwald et al. 2010), the methods did not yield an active enzyme. Only a very small number of reports describe active LOX expression by *E. coli* (Jung et al. 2003; Herwald et al. 2010; Lopez and Greenaway 2011; Smith et al. 2016), and in these cases, the characterisation of the purified enzyme is limited to activity assays and simple dye-binding assays to identify the presence of the LTQ cofactor and/or copper. In the majority of cases, the LOX has been refolded after solubilisation under denaturing conditions. The only exception to this is a report that claims LOX fusion proteins can be expressed in *E. coli* in a soluble form and purified under non-denaturing conditions in an active form (Smith et al. 2016). With a similar approach, we were able to purify a soluble form of lcp_LOX with an N-terminal MBP tag. However, this soluble MBP-lcp_LOX protein had no evident LOX activity. 

All studies that present detailed characterisation of recombinant LOX-like proteins have done so using mammalian (Zhang et al. 2018) or insect (Xu et al. 2013) expression systems where glycosylation is possible. There is evidence that the glycosylation of the pro-region could be involved in the correct folding and activity of the mature protein, and thus, its absence could be responsible for the inactivity of the recombinant barnacle lcp_LOX (Grimsby et al. 2010). Copper was included in the production of the recombinant enzyme and was not limiting. Given that this specific LOX evolved, it seems, for the purpose of interacting with cement gland proteins, it should be a priority for future studies to optimise recombinant production of the authentic lcp_LOX in systems that allow authentic post-translational modification and, thus, enable investigation of the specific functionality of this enzyme in the context of barnacle adhesion. In the absence of Lcp_LOX, the experiments proceeded using a commercial human LOXL3.

### Nonspecific Interactions of Recombinant Cement Gland Proteins with Surfaces

Before specific interactions between LOXL3, lcp3_36k_3B8 and lcp2_57k_2F5 were investigated, it was necessary to evaluate the general nonspecific adsorption characteristics of lcp3_36k_3B8 and lcp2_57k_2F5 to surfaces. The SPR-based approach has previously highlighted a possible adhesive role for the barnacle settlement-inducing protein complex (SIPC) in the temporary adhesion of barnacle cyprids, used during initial surface exploration (Petrone et al. 2015). The SIPC was found to have nonspecific surface adsorption similar to that of fibrinogen, a positive standard protein used in those studies. The recombinant lcp3_36k_3B8 and lcp2_57k_2F5 proteins did not show notable surface affinity. Increased adsorption of lcp3_36k_3B8 to a quaternary amine SAM (Fig. 3) was noteworthy and may indicate a specific affinity of the protein to amines. Since the pI of lcp3_36k_3B8 (11.9) suggests a positive net charge in the experimental conditions and, thus, no coulombic attraction between that protein and the positively charged surface, another explanation is required. Of course, the net basic pI of the protein does not preclude the existence of specific surface-exposed negatively charged domains that could interact in more complex ways with positively charged surface groups. Figure 1 highlights the presence of some isolated negative charges in the primary structure of lcp3_36k_3B8 (e.g. around 340 AA), although structural studies would be needed to identify their location in the secondary/tertiary structure. This question may indeed be moot in the present context, since the adsorption of the lcp3_36k_3B8 on the NMe_3_^+^ SAM reached only half that of fibrinogen. It should be noted, however, that the SPR response data in Fig. 3 may underestimate the binding of lcp3_36k_3B8 to the NMe_3_^+^ SAM, relative to the other interactions presented, since the sensorgrams for that protein/surface combination did not reach saturation by the end of the 3-min injection period (Supplemental 3). A structural change may have been occurring on the surface, leading to sensorgrams that began to level off before increasing again towards the end of the injection. Significant material was then lost during the final wash step.

Perhaps a more interesting observation was the negligible quantity of lcp3_36k_3B8 adsorbed to the hydrophobic SAM. Typically, a hydrophobic CH_3_ SAM is included as a positive standard surface for adsorption, with most proteins accumulating in large quantities at such a surface in an aqueous buffer, based on purely physical principles. In a highly polar solution, organic molecules, being less polar, will tend to passively accumulate at the interface between the non-polar surface and the medium, being retained there by hydrophobic interactions with the surface. This should have been true for lcp3_36k_3B8 which is, itself, hydrophobic (Table 1). The possibility that low surface binding on the CH_3_ SAM was due to residual detergent cannot be entirely eliminated, but is considered unlikely. Successful purification of the lcp3_36k_3B8 protein required the mild detergent LDAO to supress non-specific interactions with Ni–NTA beads. The electrostatically neutral LDAO detergent was removed on a desalting column prior to characterisation experiments, and the presence of trace detergent could not explain the stronger adhesion of lcp3_36k_3B8 to the quaternary amine compared to the carboxylate surface.

lcp3_36k_3B8 is contained in the α cells of the cyprid cement gland (K. Okano et al. in preparation), the contents of which are thought to constitute the central core of the cyprid cement plaque in its final morphology (Gohad et al. 2014). The plaque is covered by a lipid-rich material secreted from the β cells. It is tempting to interpret the hydrophobicity and weak interactions of lcp3_36k_3B8 with CH_3_ groups in the context of a protein present beneath and, presumably, protected by a lipidic layer in vivo. Substantial further research would be required to draw such a connection, however. In summary, the SPR data suggest that neither of the recombinant proteins exhibited significant nonspecific adsorption to any surface, but that lcp3_36k_3B8 could be resistant to nonspecific adsorption to hydrophobic materials and may have some specific affinity to amines. Of course, in this initial study, standard protein production and characterisation conditions were used—i.e. physiological pH and salt concentration. Subsequent experiments will explore the native storage conditions in the cement gland and use seawater conditions for experiments, where interactions could be different.

### Activity of LOXL3 on the Recombinant Cement Gland Proteins

The surface interaction results provided no cause to reject the motivating hypothesis of this study; that lcp2_57k_2F5 could constitute a substrate for lcp_LOX, facilitating crosslinking and curing of the cyprid cement. Consistent with this hypothesis, an in vitro LOX activity assay demonstrated the suitability of recombinant lcp2_57k_2F5 as a LOXL3 substrate, compared to BSA, which had a similar number of lysine residues per molecule but was not oxidised in the assay (Fig. 5A). Although clearly evidenced in Fig. 5A, the oxidation of lcp2_57k_2F5 by LOXL3 resulted in relatively few putative dimers and trimers (Fig. 5B) and the reasons for this will require further investigation. Also, no bands were observed with sizes that indicated formation of oligomers larger than trimers, although it is possible that if larger molecules were produced, they would not have migrated onto the SDS-PAGE gels. As to why more lcp2_57k_2F5 was not converted into dimers and trimers, possibly either the oxidation efficiency or subsequent crosslinking dynamics was responsible. It is also possible that sufficient differences exist between the human LOXL3 and native barnacle LOX to explain inefficient oxidation of the lcp2_57k_2F5 substrate in vitro. Indeed, human LOXL3 demonstrated superior activity to human LOXL2 in our initial trials (data not shown). It could also be that the experimental conditions were not optimal for oxidation of lcp2_57k_2F5 or its subsequent crosslinking. Notably, it has proven difficult to faithfully reproduce in vitro the crosslinking activity of human LOXL2 toward elastin (Schmelzer et al. 2019). Modifying crosslinking conditions for lcp2_57k_2F5 in a hypothesis-driven manner will require more detailed knowledge of the pH, redox conditions and e.g. trace metal composition of the cement glands. So, although the aim of future work should be to develop more efficient production and reaction conditions for lcp2_57k_2F5 and Lcp_LOX, it should be noted that the information required to optimise those conditions can only be derived from basic investigation of the natural system (Davey et al. 2021).

The surprising result from this study was the apparent role, in vitro, of lcp3_36k_3B8 as a LOX inhibitor (Fig. 5). The inhibitory activity of the lcp3_36k_3B8 was clear when included in the 57 kDa:LOX reaction (Fig. 5) but also appeared to be somewhat specific, having no inhibitory effect on oxidation of the model LOX substrate cadaverine. In vivo, lcp3_36k_3B8 was stored, along with LOX, in the α cells of the cyprid cement gland (K. Okano et al. in preparation). lcp2_57k_2F5, on the other hand, was localised to the β cells. It is therefore plausible that the physiological role of lcp3_36k_3B8 could be to control LOX activity. Specific investigation of competition between the lcp3_36k_3B8 and 57 kDa proteins in the presence of LOX could shed light on this. The mechanism of action of lcp3_36k_3B8, in impeding the oxidation of lcp2_57k_2F5 by LOX, is presumably via binding to the substrate (lcp2_57k_2F5) rather than by blocking the active site of the enzyme, since LOX oxidation of cadaverine was observed in the presence of lcp3_36k_3B8 (Fig. 6).

Although many details remain unclear, it seems reasonable to propose crosslinking of lcp2_57k_2F5 by LOX as a possible curing mechanism in barnacle cyprid cement on the basis of these data. This could explain cohesion of the adhesive bulk, but leaves the mechanism of adhesion to surfaces as a subject for further investigation. The predominance of β-sheet secondary structure in lcp2_57k_2F5 (Fig. 4B) also supports a structural role, perhaps via the formation of amyloid fibrils. Interestingly, the particular sensitivity of cysteine-rich proteins to sonication lysis, as was observed for lcp2_57k_2F5, has been reported previously (Stathopulos et al. 2004) and correlates with the formation of amyloid. The adult adhesive of barnacles appears fibrillar when visualised using atomic force microscopy (Sullan et al 2009), and experiments using infrared spectroscopy detected signatures of amyloid cross-β-sheet in the adult adhesive (Barlow et al. 2009, 2010). In fact, it was predicted that as much as 40% of the total secondary structure in the adhesive of adult *Amphibalanus amphitrite* could be β-sheet. Functional amyloid fibrils assemble non-covalently from proteins with substantial β-sheet content and have outstanding mechanical and biochemical stability (Fukuma et al. 2006; Smith et al. 2006; Fowler et al. 2007; Knowles and Buehler 2011), being widely recognised as components of extracorporeal protein-based materials, particularly adhesives (Mostaert et al. 2006, 2009). In the case of the cyprid adhesive, a hypothetical model could involve LOX-mediated covalent crosslinking of lcp2_57k_2F5 into oligomers that then self-assemble into amyloid fibrils, producing a robust and resistant adhesive bulk. As well as providing useful compositional detail to help understand the cyprid adhesive system, the presence of amyloid in cyprid cement would also present a structural commonality between the larval and adult adhesion systems of barnacles to complement the broad compositional similarity previously observed, in terms of a lipid-rich, phase-separating fluid at the seawater interface (Fears et al. 2018), and the presence of LOX in both cases.

### Future Perspectives

In most commonly studied taxa, the external surface of the animal is directly adhered to the substratum, including adult barnacles where the basal cuticle is attached. In these cases, it would be surprising, perhaps, if adhesion were not linked to the formation of the external surface of the animal and the inherent complexity of biological adhesives (Davey et al. 2021) may stem from their origins in such physiological processes. Identifying which processes were adopted, and how they were modified for adhesion, may prove to be the key to determining functional requirements and mechanisms of biological adhesion. For adult barnacles, a connection to the moulting cycle has been observed in vivo (Fears et al. 2018). In the case of barnacle larvae, it seems likely that their adhesion is linked to, or derived from, the process of cuticle formation. Indeed, this possibility was first mooted for adult barnacles by Thomas (1944) who proposed that, “The cement is identical in properties with the cuticle and cement glands are regarded as modified tegumental glands”. The role of LOX in ECM formation is well-established and its presence in the adhesives of both larval and adult barnacles is probably not coincidental. Therefore, in barnacles and other taxa, progress in understanding adhesion mechanisms could perhaps be accelerated by identifying origins of the adhesive precursors in their other structural materials.

## Supplementary Information

Below is the link to the electronic supplementary material.

Supplementary file1 (DOCX 18 KB)

Supplementary file2 (DOCX 15 KB)

Supplementary file3 (DOCX 157 KB)

## Data Availability

Sequences have been deposited at the DNA Data Bank of Japan, and are cited in the text.
