# Correction to: In Vitro Oxidative Crosslinking of Recombinant Barnacle Cyprid Cement Gland Proteins

**DOI:** 10.1007/s10126-022-10107-1

**Published:** 2022-03-26

**Authors:** Robert Cleverley, David Webb, Stuart Middlemiss, Phillip Duke, Anthony Clare, Keiju Okano, Colin Harwood, Nick Aldred

**Affiliations:** 1 School of Natural and Environmental Sciences, Newcastle University, Newcastle upon Tyne, NE1 7RU UK; 2 Biosciences Institute, Newcastle University, Newcastle upon Tyne, NE2 4AX UK; 3 Defence Science and Technology Laboratory, Dstl Porton Down, Salisbury, SP4 0JQ UK; 4 Department of Biotechnology, Akita Prefectural University, Akita, Japan; 5 School of Life Sciences, University of Essex, Wivenhoe Park, Colchester, CO4 3SQ UK

## Correction to: Marine Biotechnology https://doi.org/10.1007/s10126-021–10076-x

The original version of this article unfortunately contained mistakes in the author byline and in the online citation. The authors’ given and surnames were interchanged, in which, the surnames have been initialized in the citation online. The correct author names are shown above in the author byline.

The original article has been corrected.

