# Supplementary material for: In Vitro Oxidative Crosslinking of Recombinant Barnacle Cyprid Cement Gland Proteins
Source: Mar Biotechnol (NY). 2021 Oct 29;23(6):928–42. doi: 10.1007/s10126-021-10076-x (PMC8639568; doi:10.1007/s10126-021-10076-x)
Supplement: Supplementary file 1 — Supplementary file1 (DOCX 18 KB) [file 10126_2021_10076_MOESM1_ESM.docx]

**lcp3_36k_3B8 CODON OPTIMISED**

atgacatactctcgtgtttctccggttggcggctctttctacggctctgttgttcgtccg

 M  T  Y  S  R  V  S  P  V  G  G  S  F  Y  G  S  V  V  R  P

acacatccgatcatcggcacaacagttccggctacatctctgctggatggcctgctgctg

 T  H  P  I  I  G  T  T  V  P  A  T  S  L  L  D  G  L  L  L

ccggctgatatctctccggctatccgtctgatccgtacacgttacggctctctgtctcgt

 P  A  D  I  S  P  A  I  R  L  I  R  T  R  Y  G  S  L  S  R

ctggctgctatcgatgttctgcgtatcttcctgtctcgtccgcatccgctgctgctgcgt

 L  A  A  I  D  V  L  R  I  F  L  S  R  P  H  P  L  L  L  R

tctctgcaactgcgttctgttccggttgttgctgaacgtctgggcttcctgcgtcgtgct

 S  L  Q  L  R  S  V  P  V  V  A  E  R  L  G  F  L  R  R  A

atcacagctctgccgccggttttctacaacggcggctactcttctgctgttgctacatac

 I  T  A  L  P  P  V  F  Y  N  G  G  Y  S  S  A  V  A  T  Y

atccgtcaactgggcatctctatcacatcttctgctctggtttctccgctggctctgatc

 I  R  Q  L  G  I  S  I  T  S  S  A  L  V  S  P  L  A  L  I

gatgctcgtctggcttgctcttggatcgctccggtttctgttccgacattcgttgatttc

 D  A  R  L  A  C  S  W  I  A  P  V  S  V  P  T  F  V  D  F

ttcggcaaccgtatcctgcaactgggcggcggccaactggttggcggcctgccgccgctg

 F  G  N  R  I  L  Q  L  G  G  G  Q  L  V  G  G  L  P  P  L

gaaacattctacaaccgtctgacatctctgcgtttcacaccggttccggttggctttcag

 E  T  F  Y  N  R  L  T  S  L  R  F  T  P  V  P  V  G  F  Q

cgtgtaagcatcgttgctccgacagttgttcgtacactgcgtccggctttcacacgtctg

 R  V  S  I  V  A  P  T  V  V  R  T  L  R  P  A  F  T  R  L

ggcctgacaggcatggttcaaccgaacatcgttttccgtggcctggctgcttacctgtct

 G  L  T  G  M  V  Q  P  N  I  V  F  R  G  L  A  A  Y  L  S

cgtcaaggcctgaaactgccgcaattcgttggcggcctgacacatctgcaaatcccgccg

 R  Q  G  L  K  L  P  Q  F  V  G  G  L  T  H  L  Q  I  P  P

atctcttctctgatccaagatgttccgttcgattctctgctgccgcgtctgcgtttcctg

 I  S  S  L  I  Q  D  V  P  F  D  S  L  L  P  R  L  R  F  L

ccggctacagttatccgtcaagatatcgttccgctgctggctgttcgtttccgtacactg

 P  A  T  V  I  R  Q  D  I  V  P  L  L  A  V  R  F  R  T  L

ccgcgtcgtctggttcgtacactgcatctgcgtcgtgttatcgttggctacctgtctaca

 P  R  R  L  V  R  T  L  H  L  R  R  V  I  V  G  Y  L  S  T

ctggaaatcacagaagaagttacattctctccgggctacatctctacactgctgaacggc

 L  E  I  T  E  E  V  T  F  S  P  G  Y  I  S  T  L  L  N  G

ttcgatcgttacatcctgcgtcaagctaaaggtttcaaaggccgtctgtggtgccactct

 F  D  R  Y  I  L  R  Q  A  K  G  F  K  G  R  L  W  C  H  S

atctggggcgattggtaatga

 I  W  G  D  W  -  -

**Lcp2_57k_2F5 CODON OPTIMISED**

atggctacactggttggccgtggcatccatccgaaaacacgttgctgcaaaaaaacaggc

 M  A  T  L  V  G  R  G  I  H  P  K  T  R  C  C  K  K  T  G

gctatcgaaccgctgaaaggcatcacaacactggatgcttgcgaagcttactgcaaacgt

 A  I  E  P  L  K  G  I  T  T  L  D  A  C  E  A  Y  C  K  R

ctgaacctgcaatctatcaaactgggcccttcttctttcgcttgcaaacgtcctaaatac

 L  N  L  Q  S  I  K  L  G  P  S  S  F  A  C  K  R  P  K  Y

gcttgcgatcaaacatctacaggcgctggctgcgctgcttgctacaaagctggcacatgc

 A  C  D  Q  T  S  T  G  A  G  C  A  A  C  Y  K  A  G  T  C

ttcctgatgcctttcgttggcggcatccgtgtttctacacgtacagatatctctaaacgt

 F  L  M  P  F  V  G  G  I  R  V  S  T  R  T  D  I  S  K  R

cgtctggctgaacaagaaaaacgtgaacgtgttttcaaagaaaaaggcgaaggcacacgt

 R  L  A  E  Q  E  K  R  E  R  V  F  K  E  K  G  E  G  T  R

catacaggtctcggcggcgtgagctctatcacaggccaaaaaacaggcaaaaaaacagaa

 H  T  G  L  G  G  V  S  S  I  T  G  Q  K  T  G  K  K  T  E

gatgaacgtaaagctgctgaaatcctgacaaaatctgttacaacatctctgaacacagaa

 D  E  R  K  A  A  E  I  L  T  K  S  V  T  T  S  L  N  T  E

aactttggcggcttcgctaaaaacggccctgttggcgctgctgaattcctgtctggcgct

 N  F  G  G  F  A  K  N  G  P  V  G  A  A  E  F  L  S  G  A

ggcgaacttggctctttcgctggcaaaatcggccgtaacggcgctatccaacaaacaaaa

 G  E  L  G  S  F  A  G  K  I  G  R  N  G  A  I  Q  Q  T  K

caagttcaactggctaaacgtaaacgtctgaaaaacacatctggccaagttacaaaccgt

 Q  V  Q  L  A  K  R  K  R  L  K  N  T  S  G  Q  V  T  N  R

caacaagaaaacttcgttcaagatctgggcatgctgggccctggtcagaaaggctctttc

 Q  Q  E  N  F  V  Q  D  L  G  M  L  G  P  G  Q  K  G  S  F

tcttctaaaggcacaaaaaaagaactgcgttctgaacgtgctcgtcgtgctgaaacagaa

 S  S  K  G  T  K  K  E  L  R  S  E  R  A  R  R  A  E  T  E

cgtgctaaaacagaatctaaaaaactgtctctgtctggcacagctgctcatgctttcaaa

 R  A  K  T  E  S  K  K  L  S  L  S  G  T  A  A  H  A  F  K

ggctctggcggcggccctgttgaactgctggaacaaaaacaaaacgttatcggcacagaa

 G  S  G  G  G  P  V  E  L  L  E  Q  K  Q  N  V  I  G  T  E

tctgaactgcgtgaaggcacagttatctctggcggcacaacaaaagaaaacctgcgtctg

 S  E  L  R  E  G  T  V  I  S  G  G  T  T  K  E  N  L  R  L

gctggccgtggcggcgatctggaaatcgcttctctggaacgtcgttctacaggcaaagct

 A  G  R  G  G  D  L  E  I  A  S  L  E  R  R  S  T  G  K  A

aaaacacaaacagctatcaaaggccgttctctggctaaagctaaactgcgtcaagctgct

 K  T  Q  T  A  I  K  G  R  S  L  A  K  A  K  L  R  Q  A  A

acagatcaaggcctgaaccaagatctgcaaacagatgttctgggccaacgtcaaacaaac

 T  D  Q  G  L  N  Q  D  L  Q  T  D  V  L  G  Q  R  Q  T  N

acagctacacgtcagtctggctctttcgttcaactggaaaaaaaaggcaaaaaatgcgat

 T  A  T  R  Q  S  G  S  F  V  Q  L  E  K  K  G  K  K  C  D

cattgcacatctacactgaaaaaactgacaaaaggcacaacattcggctcttctcgtgaa

 H  C  T  S  T  L  K  K  L  T  K  G  T  T  F  G  S  S  R  E

cgtctgcaagaacaaacacgtcaaaaaaaacgtgatacactgcgtggccgtgctggccaa

 R  L  Q  E  Q  T  R  Q  K  K  R  D  T  L  R  G  R  A  G  Q

ggcgctacactggtttctacaggcaaagaaacaaaccgtttccgtcgtgctaaacaatct

 G  A  T  L  V  S  T  G  K  E  T  N  R  F  R  R  A  K  Q  S

ggctctggctctgatgttcaagaaaaatacaaaggcgctaactctcatctgtctacaaca

 G  S  G  S  D  V  Q  E  K  Y  K  G  A  N  S  H  L  S  T  T

ggctctgttcgtggcaaagtttctggctctgctgctacacgtacacgtaaacgttcttac

 G  S  V  R  G  K  V  S  G  S  A  A  T  R  T  R  K  R  S  Y

gaaacagaagaaggcctgtcttctcaagatgttaaagaactgaacatccttggcttccct

 E  T  E  E  G  L  S  S  Q  D  V  K  E  L  N  I  L  G  F  P

acaacaacaaaaggctaatga

 T  T  T  K  G  -  -

**lcp_LOX CODON OPTIMISED**

atgcaaaaccgtgattctttcgatttcttccgtccgccgaaccgtggccgtctgctgccg

 M  Q  N  R  D  S  F  D  F  F  R  P  P  N  R  G  R  L  L  P

ccgtctcgtcgtctggatgtttctggcgttcgtggcctgcgtacagatttccgttctaac

 P  S  R  R  L  D  V  S  G  V  R  G  L  R  T  D  F  R  S  N

aacttcatcaacttcttcggccaacgtccgtacgaaatccaactgcgtggctctaacaac

 N  F  I  N  F  F  G  Q  R  P  Y  E  I  Q  L  R  G  S  N  N

tctcgtgaaggcaacgttgaagttctgttcttcgattggcgtgaactggttgctggctgg

 S  R  E  G  N  V  E  V  L  F  F  D  W  R  E  L  V  A  G  W

cgttctgtttgcgattacggctggacaacagaacatgctaaagctgtttgccgtcaactg

 R  S  V  C  D  Y  G  W  T  T  E  H  A  K  A  V  C  R  Q  L

ggcttcccgggcaacgctgttgctacacataatggccgtttcggcatgcgttctaacggc

 G  F  P  G  N  A  V  A  T  H  N  G  R  F  G  M  R  S  N  G

atgggctctatggttaaccaatgccaaatcggcgaaggcaaccgtggcctgcgtaactgc

 M  G  S  M  V  N  Q  C  Q  I  G  E  G  N  R  G  L  R  N  C

ctgcatgctggcctgggcacagctgctgctacatgcggctctaacaacatcgctggcgtt

 L  H  A  G  L  G  T  A  A  A  T  C  G  S  N  N  I  A  G  V

atctgcggcgatgatccggcttctccgtacaacggccgtatggctgttcgtctgcgtggc

 I  C  G  D  D  P  A  S  P  Y  N  G  R  M  A  V  R  L  R  G

ggcggcacaaaaggcgatatcgaagttaaatacggcgatcgtggctggggcccgatctgc

 G  G  T  K  G  D  I  E  V  K  Y  G  D  R  G  W  G  P  I  C

ggcgatggcttcgatctgaaagatggcacagttgtttgcaaacaactgaacctgggcgct

 G  D  G  F  D  L  K  D  G  T  V  V  C  K  Q  L  N  L  G  A

gctaaacgtacatctatctctggccgtcgtgctcgtggcccgttcatcctggctggcgtt

 A  K  R  T  S  I  S  G  R  R  A  R  G  P  F  I  L  A  G  V

gaatgcacaggccgtgaagctaacctggctcaatgcaaatctatccgtgatgatccggtt

 E  C  T  G  R  E  A  N  L  A  Q  C  K  S  I  R  D  D  P  V

tcttgcccgggcaaccgttactctggcgctgctgttgaatgcacaggcccggaagatatc

 S  C  P  G  N  R  Y  S  G  A  A  V  E  C  T  G  P  E  D  I

cgtctgccggatctgcgtgttgatgctggcgaagttcaagcttctgctcgtctgtctaca

 R  L  P  D  L  R  V  D  A  G  E  V  Q  A  S  A  R  L  S  T

gaacgtctggctgatctgacatgcgctgctgaagaaaactgcctggctcgttctgcttct

 E  R  L  A  D  L  T  C  A  A  E  E  N  C  L  A  R  S  A  S

gaagttatgcgtacagatcgtaactggaaactgcgtacacgtaaactgttccgttttaca

 E  V  M  R  T  D  R  N  W  K  L  R  T  R  K  L  F  R  F  T

aacaaagtttggaacaacggcggcgctgattacaaaccgaaagctgatccggctcaatgg

 N  K  V  W  N  N  G  G  A  D  Y  K  P  K  A  D  P  A  Q  W

gaatggcatacatgccatgaacattaccattctgaagaatctttctctgaatacgatctg

 E  W  H  T  C  H  E  H  Y  H  S  E  E  S  F  S  E  Y  D  L

acatacgctggcacagatgaaaaagctgctgaaggccataaagcttctttctgcctggaa

 T  Y  A  G  T  D  E  K  A  A  E  G  H  K  A  S  F  C  L  E

gattctgaatgcaaacgtggcatctctcaacgttacttctgctacatcgaacaaaacgtt

 D  S  E  C  K  R  G  I  S  Q  R  Y  F  C  Y  I  E  Q  N  V

cgtccgccgcaaggcatccgtgctggctgcgctgatatctacggcgataacatcgattgc

 R  P  P  Q  G  I  R  A  G  C  A  D  I  Y  G  D  N  I  D  C

caatggatcgatgttacagatatcaaatctggccgttacgttctgcgtatccgtgttaac

 Q  W  I  D  V  T  D  I  K  S  G  R  Y  V  L  R  I  R  V  N

gctgatcgtaaagttcctgaagtttctttcgatgataaccaagttatctgcaacgttcgt

 A  D  R  K  V  P  E  V  S  F  D  D  N  Q  V  I  C  N  V  R

ctgaacatggaacgtgatgaagttcgtatcacaaactgccgtaacgctccgctgtaatga

 L  N  M  E  R  D  E  V  R  I  T  N  C  R  N  A  P  L  -  -
