# Supplementary material for: In Vitro Oxidative Crosslinking of Recombinant Barnacle Cyprid Cement Gland Proteins
Source: Mar Biotechnol (NY). 2021 Oct 29;23(6):928–42. doi: 10.1007/s10126-021-10076-x (PMC8639568; doi:10.1007/s10126-021-10076-x)
Supplement: Supplementary file 2 — Supplementary file2 (DOCX 15 KB) [file 10126_2021_10076_MOESM2_ESM.docx]

**Lysyl oxidase sequences in relevant taxa**

***Megabalanus rosa* LOX-like (this paper)**

MELSRLLAALLLVAGAQAQNRDSFDFFRPPNRGRLLPPSRRLDVSGVRGLRTDFRSNNFINFFGQRPYEIQLRGSNNSREGNVEVLFFDWRELVAGWRSVCDYGWTTEHAKAVCRQLGFPGNAVATHNGRFGMRSNGMGSMVNQCQIGEGNRGLRNCLHAGLGTAAATCGSNNIAGVICGDDPASPYNGRMAVRLRGGGTKGDIEVKYGDRGWGPICGDGFDLKDGTVVCKQLNLGAAKRTSISGRRARGPFILAGVECTGREANLAQCKSIRDDPVSCPGNRYSGAAVECTGPEDIRLPDLRVDAGEVQASARLSTERLADLTCAAEENCLARSASEVMRTDRNWKLRTRKLFRFTNKVWNNGGADYKPKADPAQWEWHTC**H**E**H**Y**H**SEESFSEYDLTYAGTDEKAAEGHKASFCLEDSECKRGISQRYFCYIEQNVRPPQGIRAGCADIYGDNIDCQWIDVTDIKSGRYVLRIRVNADRKVPEVSFDDNQVICNVRLNMERDEVRITNCRNAPL

***Balanus amphitrite* LOX-like (NCBI Accession: AQY78507.1)**

MDPRTLLIVTLLAVAASGQSFRDLFRDIFGSLSRPGNSTSGGGTRTSFWYPEFTSLFGQRQFSVRLVGGSNSREGNVEVLIYGSRGVVGGWRSVCDIGWTTAHAEAVCRQLGFPGPAVATRHGRFGFRSTGTGTMVSNCQVGAGGRGLRDCFYRGVGTTEGSCNSDNVAGVICGPDPSSPYGGRMDVRLRGGGTRGDVEVRYGGRGWGPVCGDGFDVKDAMVVCRQLSLGAAKKSYISGRRASGEFILAGVECSGRETSLAQCRSERGDSVRCAGNQFSGAAVECAGQQSRDLPDLRVDAQELQDSAILTTETLGDLECALDENCLAKTAAEIKRREPTLWRTRTRKLFRFTNKVWNNGKADYRPRADPSQWEWHAC**H**E**H**Y**H**SEESFSEYDLTYEGTDTKAAQGHKASFCLEDSECRRGITQKYQCYIERGTRPPQGIRAGCADIYGSYIDCQWIDVTDIRSGKYVLRVRINADRKVPEVSFDDNQVICSVDLNLEDETVRVTNCRNAPL

Likely Copper Binding Site (H-H-H)

Possible Calcium Binding sites

**Human hLOXL2 (NCBI Accession: 5ZE3_B)**

MERPLCSHLCSCLAMLALLSPLSLAQYDSWPHYPEYFQQPAPEYHQPQAPANVAKIQLRLAGQKRKHSEGRVEVYYDGQWGTVCDDDFSIHAAHVVCRELGYVEAKSWTASSSYGKGEGPIWLDNLHCTGNEATLAACTSNGWGVTDCKHTEDVGVVCSDKRIPGFKFDNSLINQIENLNIQVEDIRIRAILSTYRKRTPVMEGYVEVKEGKTWKQICDKHWTAKNSRVVCGMFGFPGERTYNTKVYKMFASRRKQRYWPFSMDCTGTEAHISSCKLGPQVSLDPMKNVTCENGLPAVVSCVPGQVFSPDGPSRFRKAYKPEQPLVRLRGGAYIGEGRVEVLKNGEWGTVCDDKWDLVSASVVCRELGFGSAKEAVTGSRLGQGIGPIHLNEIQCTGNEKSIIDCKFNAESQGCNHEEDAGVRCNTPAMGLQKKLRLNGGRNPYEGRVEVLVERNGSLVWGMVCGQNWGIVEAMVVCRQLGLGFASNAFQETWYWHGDVNSNKVVMSGVKCSGTELSLAHCRHDGEDVACPQGGVQYGAGVACSETAPDLVLNAEMVQQTTYLEDRPMFMLQCAMEENCLSASAAQTDPTTGYRRLLRFSSQIHNNGQSDFRPKNGRHAWIWHDC**H**R**H**Y**H**SMEVFTHYDLLNLNGTKVAEGHKASFCLEDTECEGDIQKNYECANFGDQGITMGCWDMYRHDIDCQWVDITDVPPGDYLFQVVINPNFEVAESDYSNNIMKCRSRYDGHRIWMYNCHIGGSFSEETEKKFEHFSGLLNNQLSPQ

Lysyl Oxidase Like Domain

Copper Binding Site (H-H-H)

Calcium Binding sites

Signal Peptide
