# Supplementary material for: In Vitro Oxidative Crosslinking of Recombinant Barnacle Cyprid Cement Gland Proteins
Source: Mar Biotechnol (NY). 2021 Oct 29;23(6):928–42. doi: 10.1007/s10126-021-10076-x (PMC8639568; doi:10.1007/s10126-021-10076-x)
Supplement: Supplementary file 3 — Supplementary file3 (DOCX 157 KB) [file 10126_2021_10076_MOESM3_ESM.docx]

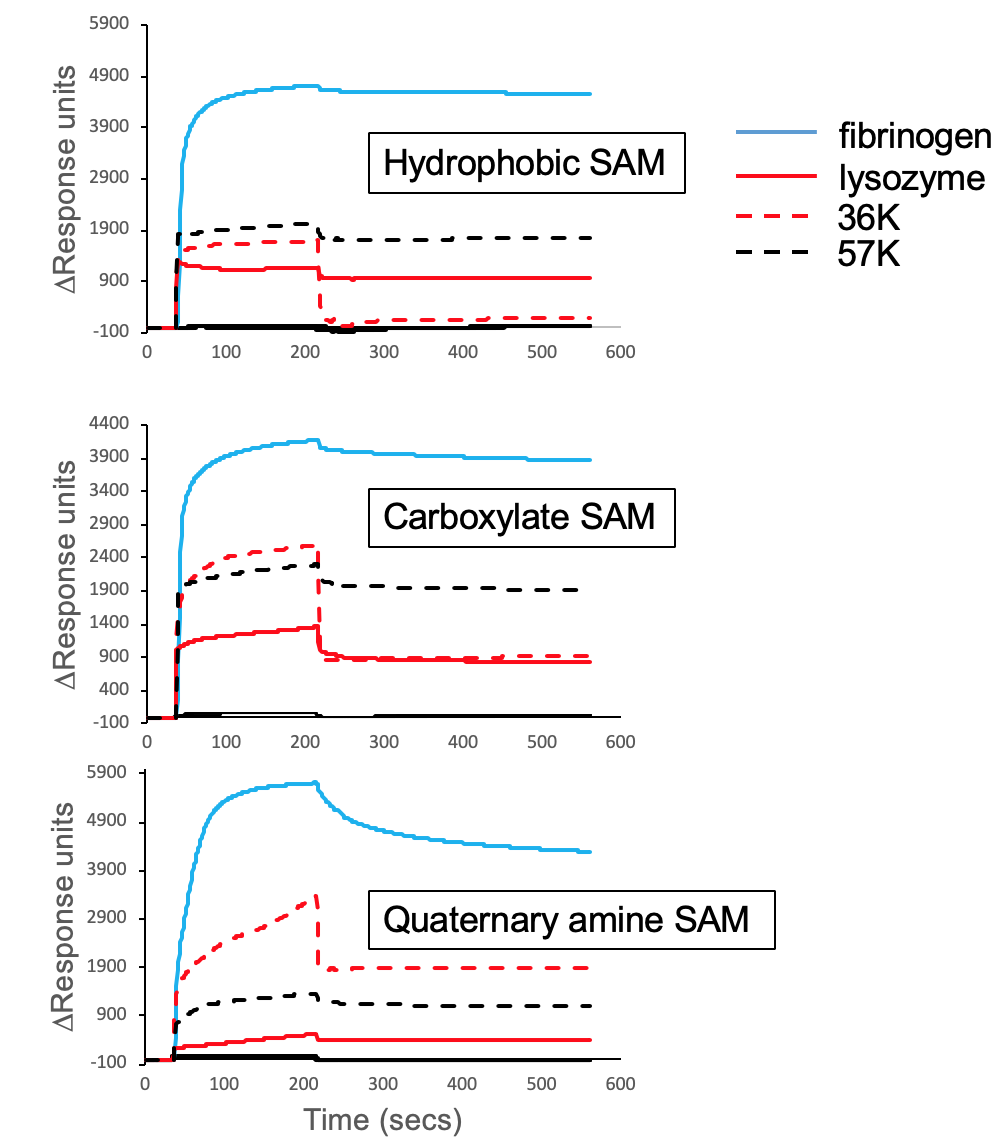


**Supplemental 3:** Representative sensorgrams for the range of four proteins adsorbing to three different self-assembled monolayers. Blue arrow indicates the unusual shoulder on the sensorgrams recorded for 36 kDa on the quaternary amine SAM, where it failed to saturate the surface during the injection period.
